# Vitamin D may influence disease course in PIMS‐TS/MIS‐C: An observational cohort study

**DOI:** 10.1111/ped.70241

**Published:** 2025-10-16

**Authors:** James E. G. Charlesworth, Joshua Navarajasegaran, Akhila Kavirayani, Avishay Sarfatti, Satish Adwani, Stéphane Paulus, Shelley Segal, James J. Gilchrist, Dominic F. Kelly, Emily A. Lees

**Affiliations:** ^1^ Department of Paediatrics Oxford University Hospitals NHS Foundation Trust, John Radcliffe Hospital Oxford UK; ^2^ Department of Paediatrics University of Oxford, John Radcliffe Hospital Oxford UK; ^3^ Acute General Medicine Oxford University Hospitals NHS Foundation Trust, John Radcliffe Hospital Oxford UK; ^4^ Paediatric Rheumatology Oxford University Hospitals NHS Foundation Trust, John Radcliffe Hospital Oxford UK; ^5^ Paediatric Intensive Care Unit Oxford University Hospitals NHS Foundation Trust, John Radcliffe Hospital Oxford UK; ^6^ Paediatric Cardiology Oxford University Hospitals NHS Foundation Trust, John Radcliffe Hospital Oxford UK; ^7^ Paediatric Infectious Diseases Oxford University Hospitals NHS Foundation Trust, John Radcliffe Hospital Oxford UK; ^8^ Oxford Vaccine Group University of Oxford, Churchill Hospital Oxford UK; ^9^ Fitzwilliam College Cambridge UK

**Keywords:** COVID‐19, MIS‐C, pediatric infectious disease, PIMS‐TS, SARS‐CoV‐2

## Abstract

**Background:**

Pediatric multisystem inflammatory syndrome temporally associated with SARS‐CoV‐2 (PIMS‐TS) remains an enigmatic disease process, with phenotypic similarities to Kawasaki disease, although many patients present with transient cardiac dysfunction. Biomarkers help validate diagnosis, but the correlation of biomarkers to disease severity or prognosis is poorly understood.

**Design:**

We retrospectively reviewed PIMS‐TS patients treated in Oxford between May 2020 and May 2022. Data on demographics, presenting features, biochemical markers, treatment, and outcomes were reviewed, and patients with/without the need for vasoactive medications were compared.

**Results:**

We identified 63 patients, median age 10.3 years (range 1.2–15.2 years). Where tested, 51/54 (94.4%) were SARS‐CoV‐2‐antibody positive. Admissions followed regional peaks in SARS‐CoV‐2 infection. Forty children (63.5%) required vasoactive medications. Amongst those requiring vasoactive medications, peak NT‐pro‐BNP (median 11,363 ng/L vs. 3741 ng/L, *p* = 0.004) and length of stay (median 8.4 vs. 6.4 days, *p* = 0.021) were greater. Vitamin D levels inversely correlated with peak CRP (spearman *r* = −0.34, *p* = 0.007) and duration of vasoactive medications (spearman *r* = −0.34, *p* = 0.030). Furthermore, low serum vitamin D correlated with lower SARS‐CoV‐2 anti‐nucleocapsid titers (spearman *r* = 0.43, *p* = 0.0014). Children receiving IV methylprednisolone, 54/63 (85.7%) (of whom, 16/54 received IVIg and methylprednisolone) had a more rapid fall in CRP than those given IVIg alone (7/63; 11.1%) or no immunomodulatory treatment (2/63; 3.2%). No patients had coronary artery aneurysm or persistent cardiac sequelae at discharge.

**Conclusions:**

Methylprednisolone suppressed CRP early and without evidence of coronary aneurysm in this cohort. We demonstrate a relationship between vitamin D, SARS‐CoV‐2 anti‐nucleocapsid antibody production, inflammatory biomarkers, and duration of vasoactive medication, requiring further validation.

## INTRODUCTION

Pediatric Inflammatory Multisystem Syndrome Temporally Associated with SARS‐CoV‐2 (PIMS‐TS), otherwise known as Multisystem Inflammatory Syndrome in Children (MIS‐C), was first identified in June 2020.^1^ Cases typically occur 2 to 6 weeks following SARS‐CoV‐2 infection and have a male preponderance.^1^ PIMS‐TS incidence estimates vary between 6 and 40 PIMS‐TS cases per 100,000 SARS‐CoV‐2 infections.^2^, ^3^ This broad range likely represents differing case definitions,^4^ methodology for case ascertainment, and timing of data collection throughout the pandemic. During early 2022, UK PIMS‐TS cases fell despite much higher SARS‐CoV‐2 incidence with the Omicron variant; UK seropositivity was up to 80% in older children.^5^ This suggests PIMS‐TS requires SARS‐CoV‐2‐naïve hosts and is not driven by SARS‐CoV‐2 variants.^6^ However, PIMS‐TS cases continue to be reported with endemic SARS‐CoV‐2, despite past SARS‐CoV‐2 exposure or vaccination.^7^ Debate is ongoing as to whether PIMS‐TS is related to immune dysregulation with impaired antigen presentation and delayed viral clearance,^8^ or related to SARS‐CoV‐2 spike proteins behaving as a superantigen, inducing a hyperinflammatory state.^9^

The age range of PIMS‐TS patients is older than typical for Kawasaki disease (KD), with a median of 9 years, versus 2.7 years for KD.^1^ PIMS‐TS can cause severe disease in previously healthy children,^10^ with up to 45% of UK cases requiring Pediatric Intensive Care Unit (PICU) admission.^11^ Children of Black, Asian, and other ethnic groups are disproportionately affected in the UK (odds ratio for disease: 15.7, 4.0, and 11.2, respectively).^12^ An early UK national consensus statement suggested initial management with intravenous immunoglobulin (IVIg), with methylprednisolone as second‐line therapy.^13^

Vitamin D deficiency is common in children in England, with an estimated prevalence of 261 per 100,000 person years.^14^ Vitamin D levels have been postulated to have a role in cytokine regulation and immune response, with the vitamin D receptor widely expressed on immune cells and thought to modulate innate and adaptive immunity.^15^ Vitamin D supplementation can reduce the risk of acute respiratory tract infection.^16^ Limited data support vitamin D sufficiency in reducing mortality rates in SARS‐CoV‐2 infection in adults,^17^ postulated to be by reduction of cytokine storm (with CRP used as a proxy measure). A small UK study noted that 78% (14/18) children with PIMS‐TS were vitamin D deficient, with a non‐significantly lower level in children who required PICU support.^18^ Given the suggestion that PIMS‐TS represents a hyperinflammatory state and/or immune dysregulation, the role of vitamin D levels in this condition warrants further investigation, as it has not been widely reported amongst PIMS‐TS/MIS‐C patients.

In this article, we describe the experience of PIMS‐TS in our tertiary center, outlining demographics, clinical and biochemical features, therapeutic management, and early outcomes for cases presenting over a 25‐month period.

## METHODS

### Study design and population

Retrospective case review of all patients aged 18 years or younger, fulfilling the RCPCH diagnostic criteria for PIMS‐TS,^4^ admitted to Oxford Children's Hospital, between May 1, 2020, and May 31, 2022. Cases were identified through referrals to pediatric infectious diseases and using electronic health records (EHR) for ICD‐10 code “U075.” Nine patients were recruited to the “Recovery” trial (https://www.recoverytrial.net); however, no patients were randomized to receive biologics; therefore, these patients' care represented standard practice in our setting. Data were extracted from EHR on patient demographics, presenting features, duration of stay, PICU management, laboratory and radiographic/echocardiology findings, immunomodulation and vasoactive medications. In addition, we completed a PubMed search for articles on PIMS‐TS/MIS‐C in English, reporting on presenting symptoms, clinical and laboratory outcomes, and treatments. This yielded 30 studies with case series of 10 or more patients, with data across each of these categories, summarized in Table S1.

### Analysis and statistics

We compared PIMS‐TS admissions over time to SARS‐CoV‐2 reported case rates for Oxfordshire aged <20 years over the same period. This dataset was obtained from the UK government's COVID‐19 dashboard (https://coronavirus.data.gov.uk, accessed August 2022). All patient data were pseudonymized at the point of extraction and collated in Microsoft Excel 2013 (version 15.0.5493.1000, California, USA). All graphical data presentation and statistical analysis were performed in GraphPad Prism (version 10.1.0, California, USA). See Supplementary methods for statistical analysis.

### Ethics statement

Data collection was retrospective, pseudonymized, and collated for local quality improvement purposes. Data were fully anonymized for analysis. In keeping with UK Health Research Authority (UK HRA) advice, formal ethics review was not required as clinicians (authors of this manuscript) were recruited to report anonymized patient data and report the outcomes of multiple domains, detailed in Supplementary methods.

## RESULTS

**FIGURE 1:**
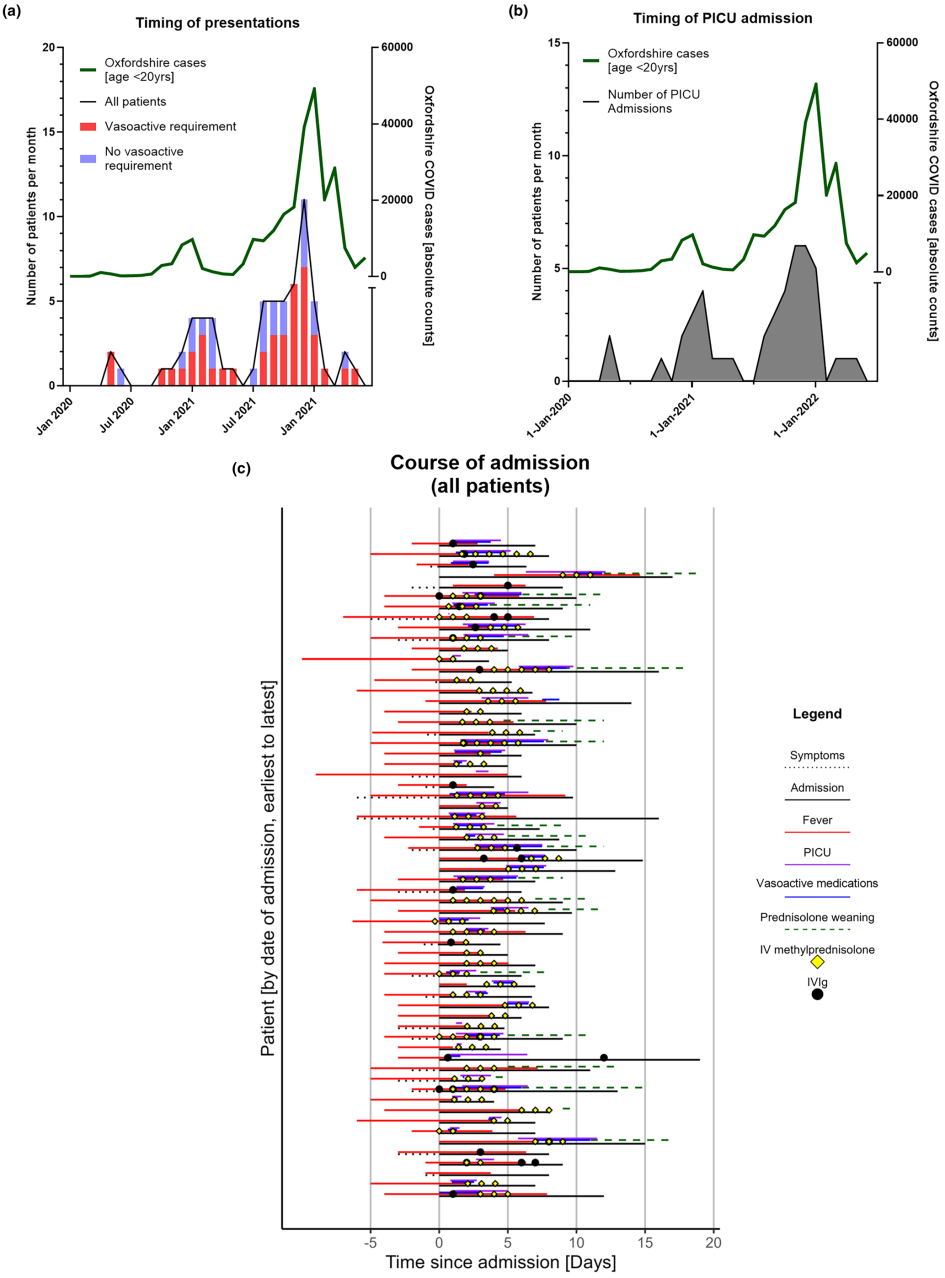
Timing of all PIMS‐TS presentations (a and b) and overview of course of all admissions (c). Absolute counts of confirmed cases of COVID‐19 for all patients aged 0 to 19 years across Oxfordshire are shown by month (right *Y* axis, a and b), for context. Absolute counts of PIMS‐TS cases (receiving vasoactives—red bars, and no requiring vasoactives—blue bars) are shown across the study period by month (left *Y* axis) (a). Absolute counts of pediatric intensive care unit (PICU) admissions alone are shown (Left *Y* axis, b). A graphical summary of individual patient admissions is shown (c). Lines represent time in days (*X* axis), showing all patients admitted with PIMS‐TS/MIS‐C ordered by date of admission (*Y* axis), earliest (top) to latest (bottom). Lines and symbols show total inpatient admission time (black lines), symptom onset/duration prior to admission (dashed black line), time with fever prior to and during admission (red line), PICU length of stay (purple line), time on vasoactives (blue line), time weaning prednisolone (dashed green line), timepoint of IV methylprednisolone (yellow diamond), and IVIg (black circle) administration.

PIMS‐TS case rates for the region closely followed SARS‐CoV‐2 peaks amongst children across Oxfordshire (Figure 1a). Median time between reported SARS‐CoV‐2 infection/exposure and presentation was 28 days (range 3–56) across groups. Eleven (17.5%) patients were unable to identify a clear SARS‐CoV‐2 contact or positive result, likely representing asymptomatic spread and the high incidence of SARS‐CoV‐2 at the time.^19^ The proportion of cases requiring vasoactive medications (Figure 1a, red bars) or PICU admission (Figure 1b) appeared consistent across the study period and aligned with community SARS‐CoV‐2 prevalence.

We identified 63 patients with PIMS‐TS, with a median age of 10.3 years (range 1.2–15.2 years) and 42.9% female. Nineteen children (30.2%) had any documented co‐morbidity, the commonest being autistic spectrum disorder (five; 7.9%), asthma (three; 4.8%), and eczema (three; 4.8%). There were 46 admissions (73%) to PICU, for a median of 2.7 days (range 0.07–6.4; one assessed for stepdown on arrival). Only five (7.9%) admissions were intubated, all of whom required cardiovascular support and presented to a district general hospital. Intubation was required to facilitate transfer with challenging fluid/cardiovascular management, and/or respiratory compromise, alongside facilitating central venous access. One case was intubated for encephalopathy.

### Patients requiring vasoactive medications had a longer length of stay and higher peak NT‐pro‐BNP

We explored factors, which may indicate a requirement for vasoactive medications, as a surrogate for disease severity (Table 1). Forty (63.5%) patients required vasoactive medications (vasopressors and/or inotropes) for cardiac dysfunction. Hypotension was the only presenting feature which was positively associated with a significant need for vasoactive medications (*p* = 0.031). Presentations which included non‐purulent conjunctivitis, cough, or joint pain were significantly less likely to need vasoactive medications (*p* = 0.039, *p* = 0.038 and *p* = 0.031, respectively). The majority (71.9%) of patients had abdominal pain, with five (7.9%) undergoing laparoscopic appendicectomy prior to a PIMS‐TS diagnosis. These patients were clinically and biochemically indistinct within the cohort.

**TABLE 1:** Summary of patient demographics, clinical management, biomarkers, and treatment received.

	Total cohort	Patients requiring vasoactive medications	Patients *not* requiring vasoactive medications	*p* value
Number (% of total)	63	40 (63.5)	23 (36.5)	–
Sex [*N* (%) female]	27 (42.9)	20 (50)	7 (30.4)	0.066^§^
Ethnicity [*N* (%)]				
White	23 (36.5)	19 (47.5)	4 (17.4)	–
Asian	5 (7.9)	2 (5.0)	3 (13.0)	–
Black	8 (12.7)	3 (7.5)	5 (21.7)	–
Mixed	2 (3.2)	2 (5.0)	0 (0.0)	–
Not known/recorded	25 (39.7)	14 (35.0)	11 (47.8)	–
Age at presentation (years)	10.3 [1.2–15.2]	10.2 [1.4–15.1]	11.9 [1.2–15.2]	0.109^§^
Weight centile (WHO centile)	74.1 [2–99]	50.5 [2–99]	81.0 [25–98]	**0.023** ^**§**^
Any co‐morbidities [*N* (%)]	19 (30.2)	11 (27.5)	8 (34.8)	**0.037** ^**§**^
Timing of COVID positive or exposure (days before presentation)	28 [3–56]	28 [3–55]	28 [5–56]	–
Timing of symptoms to presentation (days)	0 [0–6]	0 [0–6]	0 [0–5]	–
Patients with no known COVID exposure [*N* (%)]	11 (17.5)	6 (15.0)	5 (21.7)	>0.99^b^
*Presenting features*				
Lymphadenopathy [*N* (%)]	21 (32.8)	12 (30)	9 (37.5)	0.740^§^
Non‐purulent conjunctivitis [*N* (%)]	26 (40.6)	15 (37.5)	11 (45.8)	**0.039** ^§^
Maculopapular rash [*N* (%)]	42 (66.7)	30 (75)	12 (52.2)	0.125^§^
Cough [*N* (%)]	11 (17.2)	3 (7.5)	8 (33.3)	**0.038** ^§^
Joint pain [*N* (%)]	6 (10.9)	2 (5.0)	4 (17.4)	**0.031** ^§^
Oromucosal inflammation [*N* (%)]	19 (29.7)	14 (35)	5 (20.8)	0.188^§^
Vomiting [*N* (%)]	36 (56.3)	22 (55)	14 (58.3)	0.345^§^
Diarrhea [*N* (%)]	27 (42.2)	17 (42.5)	10 (41.7)	0.208^§^
Abdominal pain [*N* (%)]	46 (71.9)	30 (71.9)	16 (66.7)	0.133^§^
(appendicectomy)	(5 = 7.8%)^c^	(3 = 7.5%)	(2 = 8.3%)	(−)
Weakness [*N* (%)]	3 (4.7)	2 (5.0)	1 (4.2)	–
Confusion [*N* (%)]	2 (3.1)	2 (5.0)	0 (0)	–
Headaches [*N* (%)]	20 (31.3)	11 (27.5)	9 (37.5)	0.151^§^
Hypotension [*N* (%)]	34 (53.1)	28 (70)	6 (25)	**0.031** ^§^
Shocked [*N* (%)]	14 (21.9)	13 (32.5)	1 (4.2)	0.051^§^
*Inpatient course*				
PICU admission [*N* (%)]	46 (71.9)	40 (100)	6 (25)	**<0.001** ^b^
Length of PICU admission (days)	2.7 [0.07–6.4]	3.0 [0.4–6.4]	0.6 [0.1–1.3]	**<0.001** ^a^
Total length of hospital stay (days)	7.8 [3–19]	8.4 [3–19]	6.4 [3.6–11]	**0.021** ^a^
Patients intubated [*N* (%)]	5 (7.8)	5 (12)	0 (0)	–
*Laboratory values (peak/trough)*				
Highest CRP (μg/mL)	196.8 [48–459.3]	203.4 [83.3–459.3]	193.7 [48–331]	0.630^ǂ^
Lowest lymphocyte count (×10^9^/L)	0.77 [0.14–2.1]	0.67 [0.1–2.1]	0.77 [0.3–2]	0.346^ǂa^
Lowest platelet count (×10^9^/L)	163 [14–359]	146 [14–359]	165 [75–354]	**0.046** ^**ǂ**^
Highest ferritin	835.3	1046.0	684.4	0.160^ǂ^
(μg/L)	[164.6–10,068.8]	[328.3–10,068.8]	[164.6–5311]	
Highest NT‐Pro‐BNP (ng/L)	7879.9	11,363.1	3741.0	**0.004** ^ǂ^
	[92–77,051.8]	[972.9–77,051.8]	[92–12,827.4]	
Highest troponin (ng/L)	131.0	140.0	93.0	0.468^ǂ^
	[0–19,773]	[3–19,773]	[0–2068]	
Highest D‐dimer (ng/mL)	6500.0	6992.0	3898.0	0.840^ǂ^
	[1431–61,980]	[1431–39,050]	[1477–61,980]	
Lowest vitamin D (nmol/L)	28 [9–74]	27.5 [9–70]	29 [9–74]	0.882^ǂ^
Coronary artery aneurism (*Z* score ≥ 2.5)	Nil	Nil	Nil	–
Lowest LV ejection fraction [%]	52 [18–71]	50.0 [18–71]	60.0 [28–70]	–
COVID‐19 antibody positive	51 (94.4)	33 (94.3)	18 (94.7)	–
[*N* (%) of those tested]	9 (14.3%) not tested	5 (12.5%) not tested	4 (17.4%) not tested	
Anti‐S titer (AU/mL)	1031.5	972.25	1092.4	–
	[6.4–4847.3]	[6.4–4847.3]	[309.1–4374]	
Anti‐N titer (AU/mL)	3.91 [0–7.54]	3.81 [0.2–7.5]	5.19 [0–7.5]	–
*Treatment*				
IV methylprednisolone received [*N* (%)]	54 (85.7)	36 (90)	19 (82.6)	0.150^b^
Timing of IV methylprednisolone (days since presentation)	2.0 [0–9]	1.7 [0–9]	2.0 [0–6]	0.810^a^
IVIg received [*N* (%)]	23 (36.5)	17 (42.5)	6 (26)	0.278^b^
Timing of IVIg [days since presentation]	1.8 [0–8]	1.8 [0–8]	2.5 [0.9–5]	0.656^a^
Received both IV methylprednisolone and IVIg [*N* (%)]	16 (25)	13 (32.5)	3 (13.0)	0.133^b^
Prednisolone weaning [*N* (%)]	23 (36.5)	18 (45)	5 (21.7)	0.102^b^

*Note*: Shown for all patients and separated by need for vasoactive medications. Unless otherwise stated (number and %), values represent median [and range]. Values shown in bold highlight where *p* < 0.05. Treatment totals: *n* = 38 IV methylprednisolone alone, *n* = 7 IVIg alone, *n* = 16 IVIg and IV methylprednisolone, *n* = 2 neither IV methylprednisolone nor IVIg. *N* = 23 patients received a weaning course of prednisolone, of these *n* = 21 had received IV methylprednisolone prior and *n* = 2 received IVIg followed by weaning prednisolone. *N* = 9 patients received both IVIg and IV methylprednisolone followed by weaning prednisolone.

Abbreviations: COVID, coronavirus infectious disease (COVID‐19); CRP, C‐reactive protein; IVIg, intravenous immunoglobulin; IV, intravenous; N/A, not applicable; NT‐Pro‐BNP, N‐terminal pro‐brain natriuretic peptide; PICU, pediatric intensive care unit; reference range: sufficient >50 nmol/L, insufficient 25–50 nmol/L, deficient <25 nmol/L; Vitamin D, measured as 25‐hydroxy vitamin D.

^a^ Represents *p* values for continuous variables, compared by multiple Mann–Whitney *U* tests. Controlled for multiple testing by applying a false discovery rate of 1%, with the two‐stage step‐up method of Benjamini, Krieger, and Yekutieli; therefore, the resultant *q* value is shown.

^b^ Represents *p* values from Fisher's exact test for dichotomous outcomes. Logistic regression was used in separate analyses to determine factors which may determine a requirement for cardiovascular support. These were split into two separate analyses of presenting features (§) and laboratory results (ǂ), both controlling for sex, age, and weight centile. Non‐assessed variables have no *p* value shown (−) due to incomplete data.

^c^ *N* = 5 children underwent appendicectomy prior to PIMS‐TS diagnosis. These children had a similar age (median 12.6 years, range 8.3–15.1 years), peak CRP (median 190), and need for vasoactive medications (3/5; 60%) as the total cohort.

Patients who required vasoactive medications had a greater length of hospital and PICU admission (*p* < 0.015 and *p* < 0.001, respectively), higher peak NT‐pro‐BNP (median 11,363.1 vs. 3741.0 ng/mL, *p* = 0.013), and lower trough platelet count (median 146 vs. 165 × 10^9^/L, *p* = 0.046). Six (9.5%) patients had coronary ectasia on early echocardiograms, which resolved within five days. No patients developed coronary artery aneurysm (CAA). Greater proportions of patients requiring vasoactive medications were given methylprednisolone (90% vs. 78.3%), IVIg (42.5% vs. 26%), both methylprednisolone and IVIg (32.5% vs. 13%), and prednisolone weaning (45% vs. 21.7%), although these comparisons did not reach significance due to the small size of the cohort. Figure 1c demonstrates the time course of disease and treatment for all patients.

### Laboratory markers for PIMS‐TS admissions correlate with length of stay

**FIGURE 2:**
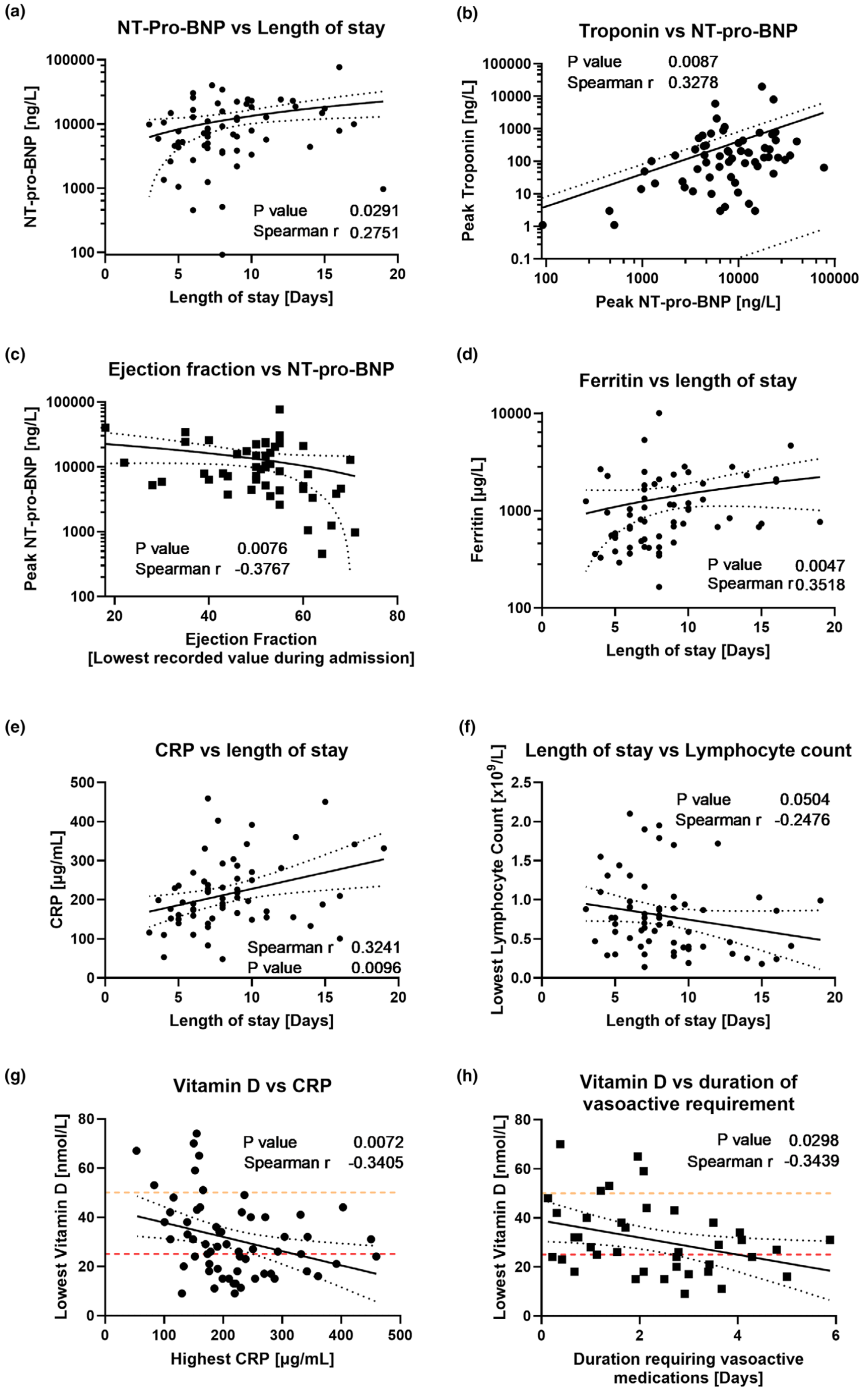
Exploratory comparison of laboratory values and demographics throughout admission. Comparison was made between NT‐pro‐BNP and length of stay (a), highest troponin (b) and lowest reported left‐ventricular ejection fraction (c). Length of stay was further compared with highest ferritin (d), highest CRP (e) and lowest reported lymphocyte count (f). Lowest recorded serum vitamin D was compared against CRP (g) and duration of time spent on vasoactive medication infusions for cardiovascular support (h). Vitamin D measured as 25‐hydroxy vitamin D throughout. UK guideline levels of vitamin D insufficiency (50 nmol/L—yellow dashed line, to 25 nmol/L—red dashed line) and deficiency (<25 nmol/L—below red dashed line) are shown. For all plots, Spearman correlation (*r* and *p* values) is shown. Line of fit results from simple linear regression with 95% confidence intervals shown.

Patients demonstrated high acute inflammatory markers (CRP, ferritin, D‐dimer), elevated cardiac enzymes (NT‐pro‐BNP and troponin), suppressed lymphocyte counts, and low serum vitamin D, measured as 25(OH)D (reference range: sufficient >50 nmol/L, insufficient 25–50 nmol/L, deficient <25 nmol/L) (Table 1). To explore associations amongst these continuous variables, we performed an exploratory correlation matrix (Figure S1). This highlighted significant correlations between peak NT‐pro‐BNP and length of stay (both PICU, *r* = 0.493, *p* < 0.001, and total hospital admission, *r* = 0.275, *p* = 0.029, Figure 2a), peak troponin (Figure 2b, *r* = 0.328, *p* = 0.009), and lowest reported ejection fraction (Figure 2c, *r* = −0.377, *p* = 0.008). Length of stay positively correlated with peak ferritin (Figure 2d, *r* = 0.352, *p* = 0.005), CRP (Figure 2e, *r* = 0.324, *p* = 0.010), and showed marginal significance with lowest lymphocyte count (Figure 2f, *r* = −0.248, *p* = 0.05). Figure S2 demonstrates correlations between age and demographic/laboratory markers. The only laboratory marker significantly correlating with peak CRP was lowest vitamin D (Figure 2g, *r* = −0.341, *p* = 0.007). Furthermore, lower vitamin D correlated with children requiring vasoactive medications for longer (Figure 2h, *r* = −0.344, *p* = 0.029). Vitamin D level was insufficient (49.2% (30/61) of those tested) or deficient (24/61; 39.3%) in the majority of children.

### SARS‐CoV‐2 serology correlated with vitamin D status

**FIGURE 3:**
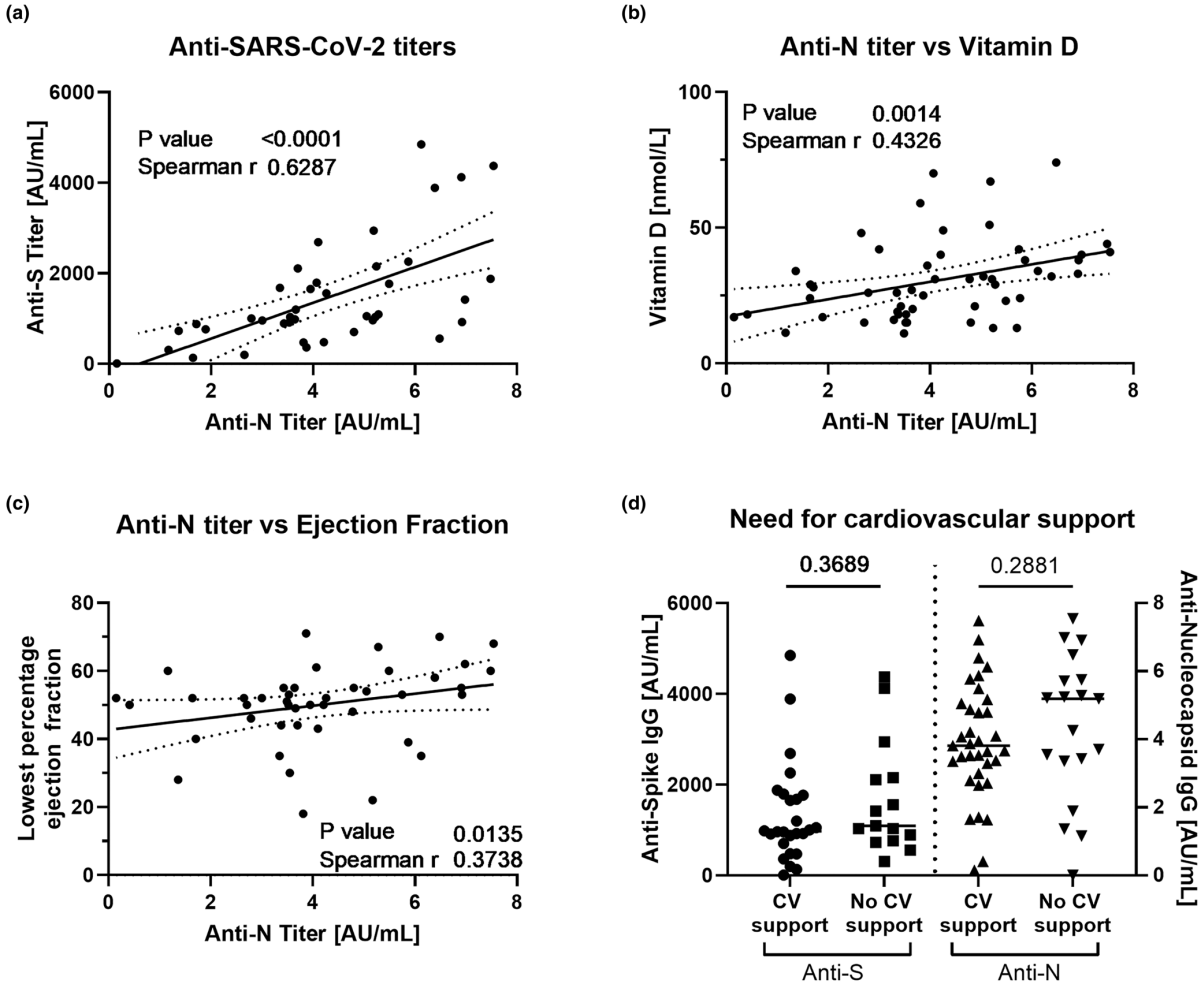
Comparison of anti‐SARS‐CoV‐2 serology with vitamin D and clinical findings. Antibody titers towards SARS‐CoV‐2 were obtained for anti‐S and anti‐N; the correlation for the two is shown in (a). Anti‐S titers were not performed for all antibody‐tested patients; therefore, relationships to anti‐N titers are explored. Anti‐N titers are shown against the lowest reported serum vitamin D (measured as 25‐hydroxy vitamin D) (b) and the lowest reported ejection fraction (c). Antibody titres are shown divided by the need for vasoactive medications (d); statistics shown are the result of the Mann–Whitney *U* test. For a–c, results of the Spearman correlation (*r* and *p* values) are shown. Line of fit results from simple linear regression with 95% confidence intervals shown.

SARS‐CoV‐2 serology was performed for 54/63 (86%) admissions; of these, 51 (94.4%) were positive. Where both spike (anti‐S) and nucleocapsid (anti‐N) antibodies were assessed, resultant titers correlated closely (Figure 3a, *r* = 0.629, *p* < 0.0001). Anti‐N was assessed routinely, with anti‐S reported in 78% (42/54) of those tested. Anti‐N titers showed a positive relationship with serum vitamin D (Figure 3b, *r* = 0.437, *p* = 0.0014), and lower anti‐N titers were associated with the lowest recorded ejection fraction (Figure 3c, *r* = 0.374, *p* = 0.014). There was no difference in the need for vasoactive medications with either SARS‐CoV‐2 antibody titer (Figure 3d).

### Suppression of CRP was most rapid with methylprednisolone

All except two admissions (61/63; 96.8%) received immunomodulation (IVIg and/or IV methylprednisolone) (Table 1, Figure 1c). Seven cases (10.9%) were treated with IVIg alone, 54 (85.7%) received IV methylprednisolone, of whom 16 (29.6%) received both IVIg and methylprednisolone. Weaning oral prednisolone was given to 23/63 cases (36.5%). No patients received biologic immunomodulators.

**FIGURE 4:**
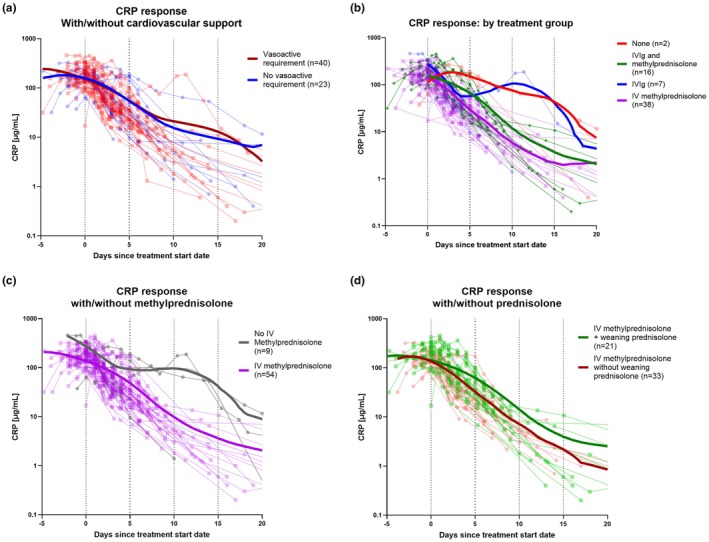
Change in CRP plotted against time since starting immunomodulatory treatment(s). Results from all available CRP values are plotted by days since the start of immunomodulatory treatment (or admission if no immunomodulatory treatment given). Fall in CRP is shown by subgroups who did (red) and did not (blue) receive vasoactive medication (a), vitamin D status (b—sufficient (green), ≥50 nmol/L, insufficient (amber) ≥25–<50 nmol/L or deficient (red) <25 nmol/L), and oral prednisolone tapering following IV methylprednisolone (c—received in green, not received in red). CRP response by immunomodulatory therapy (d) is shown for those receiving none (red), IVIg and IV methylprednisolone (green), and IV methylprednisolone alone (blue). For all datasets, individual patient CRP values are shown by points connected with lighter/thinner lines, colored by subgroup. Darker/thicker lines represent fitted curves for indicated subgroups (numbers within each subgroup shown in brackets), fitted to individualized data by lowess curve, using a coarse 5‐point smoothing window. Vitamin D is measured as 25‐hydroxy vitamin D.

All patients demonstrated a fall in CRP following the start of immunomodulatory treatment (or admission if none received, Figure 4a). Resolution of fever was similar following IV methylprednisolone (median 0.92 days, range −1.5 to 7.9) and IVIg (median 1.2 days, range −0.8 to 6.9) (Figure S3). However, patients requiring both IVIg and methylprednisolone had prolonged fever from first treatment (median 2.9 days, range −0.3 to 3.3), compared with methylprednisolone or IVIg monotherapy (*p* = 0.026 and *p* = 0.103, respectively). The two patients receiving no immunomodulation remained febrile for 3.8 and 5 days after admission. CRP response to treatment appeared similar regardless of the need for vasoactive medications, vitamin D status, or amongst those given a prednisolone weaning regimen (Figure 4a–c, respectively). The addition of oral prednisolone to the treatment regimen did not affect the decline in CRP (Figure 4c). Patients treated with IV methylprednisolone and IVIg + methylprednisolone demonstrated similar declines in CRP, whereas the seven patients receiving IVIg alone and two patients receiving no immunomodulatory therapy demonstrated elevated CRP past day 8 of admission (Figure 4d); however, this may be skewed by data paucity beyond day 7.

## DISCUSSION

We demonstrate early and sustained suppression of CRP with methylprednisolone, without evidence of CAA, in PIMS‐TS patients from our cohort. We describe novel relationships between serum vitamin D, SARS‐CoV‐2 anti‐nucleocapsid antibody titers, inflammatory biomarkers, and a prolonged need for vasoactive medication in PIMS‐TS/MIS‐C.

The demographics within our cohort fit other studies of PIMS‐TS, in terms of age, male predominance, and a lack of severe co‐morbidities^1^, ^10^, ^20^ Ethnicity was poorly recorded, so we are unable to report on previously noted disparities,^21^ or correlate vitamin D status with ethnicity (given higher rates of vitamin D deficiency amongst Black and Asian UK residents^22^). Reported rates of PICU admission vary widely from 29% to 73.8%^11^, ^21^, ^23^ suggesting our PICU‐admitted cohort is high (71.9%). This may reflect data missing from locally managed patients, not requiring transfer to our tertiary center, particularly as local experience increased throughout the pandemic. We acknowledge that the greatest limitation of our study is the retrospective observational approach, which relies on routinely obtained clinical data. There is therefore no control group(s) with similar clinical and biochemical data, and as such, these data cannot be used to discriminate PIMS‐TS/MIS‐C from other inflammatory or infective disorders.

We show lower serum vitamin D significantly correlated with higher peak CRP and lower SARS‐CoV‐2 anti‐N titers, adding to a wealth of evidence that vitamin D has an immunomodulatory role.^16^, ^24^, ^25^ A Dutch study of 923 patients with inflammatory and non‐inflammatory diseases found that greater serum vitamin D was associated with lower CRP (regression coefficient −0.879, *p* < 0.001), with a stronger effect in inflammatory disorders.^26^ In critically ill children, a pre‐pandemic meta‐analysis demonstrated children with insufficient and deficient vitamin D had significantly greater vasoactive requirements and higher mortality.^27^ In cohorts of primary SARS‐CoV‐2 infection (COVID‐19), low vitamin D status is a prognosticator for severity and mortality amongst adults.^28^ Furthermore, vaccine‐induced SARS‐CoV‐2 antibody titers are slightly, but significantly, increased amongst participants with greater baseline vitamin D.^29^ Together, the suggested poorer viral control and reduced post‐vaccination antibody titers in the context of low vitamin D states may support our observation of lower anti‐N titers. However, the immunological mechanisms underlying these responses remain poorly understood. Low vitamin D is associated with PIMS‐TS/MIS‐C disease severity internationally, although remains unreported in the majority of cohorts (Table S1). In keeping with our own findings, we identified five studies internationally (*n* = 122 Iran,^30^ *n* = 51 and *n* = 34 Turkey,^31^, ^32^ *n* = 31 USA^33^ and *n* = 21 Croatia^34^) supporting vitamin D insufficiency/deficiency to be significantly associated with severe versus mild–moderate MIS‐C, and the proportion of patients requiring cardiovascular support. Two of these case series included cohorts of age‐matched healthy controls, confirming significantly greater rates of vitamin D insufficiency/deficiency amongst MIS‐C patients.^31^, ^32^ Demonstrating vitamin D status and inflammatory activity in PIMS‐TS in our dataset remains a novel observation.

Our findings demonstrating the effective suppression of CRP (and by extrapolation, inflammation) with corticosteroids reflect findings from a retrospective study of 111 children in France with MIS‐C, noting a beneficial effect of adding corticosteroids in combination with IVIg. Fewer children had treatment failure (fever persisting 2 days after initial therapy or recrudescence within 7 days) in the methylprednisolone + IVIg group (9%) versus IVIg alone (51%).^35^ Furthermore, data from the “Overcoming COVID‐19” registry (*n* = 518 patients, USA) showed initial treatment with IVIg + glucocorticoids was associated with a significantly lower risk of cardiovascular dysfunction from day 2 versus IVIg alone (RR 0.56) and a lower likelihood of requiring further immunomodulation.^36^ The Best Available Treatment Study (BATS) collated worldwide data on 2101 patients, showing no significant difference in recovery from MIS‐C after treatment with IVIg alone, IVIg + glucocorticoids, or glucocorticoids alone, suggesting that initial glucocorticoid treatment would be a safe alternative in areas where IVIg is not accessible.^37^ In November 2021, the World Health Organization updated their guidance on the management of MIS‐C to recommend the use of corticosteroids (rather than IVIg) in addition to supportive care in MIS‐C children and corticosteroids in addition to standard care (IVIg) for those fitting MIS‐C and KD. The WHO advice reflects the treatment approach used at our center, where, following discussion in dedicated multidisciplinary meetings, children with a Kawasaki‐like phenotype received IVIg as a first‐line treatment, and those with a non‐specific PIMS‐TS/MIS‐C phenotype received methylprednisolone unless recruited to a clinical trial.

Risk of CAA with PIMS‐TS is difficult to predict; laboratory findings do not predict future development of CAA or dilatation.^1^ Rates of coronary artery abnormalities have been variable; one review noted a reported incidence of 6%–24%.^28^ In the large BATS dataset, 236/1918 (12.3%) patients had CAA, with 182/196 (92.9%) resolving under early follow up.^37^ No children in our study developed aneurysms, which may reflect the small sample size, but it is equally possible that CAA rates in PIMS‐TS are overestimated if younger patients are described as “Kawasaki‐phenotype,” given KD cases have continued to occur during the SARS‐CoV‐2 pandemic.^38^ However, early corticosteroids are also superior to IVIg alone in preventing CAA in KD.^39^ A tapering regime of corticosteroids was used in 22 of our patients, as advised by National Institutes of Health (NIH) clinical guidelines for MIS‐C, to avoid rebound inflammation.^40^ Lack of CAA in this subgroup, mainly treated with methylprednisolone alone, is reassuring; furthermore, we found no rebound CRP with or without weaning prednisolone.

In our cohort, we demonstrate the efficacy of methylprednisolone at rapidly decreasing CRP as a marker of inflammation, without apparent safety concerns in terms of increased risk of CAA, supporting the most recent BATS data.^37^ Although exploratory, our analysis reveals previously unreported correlations between biochemical markers such as serum vitamin D, CRP, and anti‐SARS‐CoV‐2 antibody titers and confirms the clinically relevant association of vitamin D status and duration of vasoactive requirement. These findings require further exploration in PIMS‐TS/MIS‐C and other systemic inflammatory disorders, and do not determine the role of vitamin D, or whether vitamin D status pre‐dates or is subsequent to PIMS‐TS/MIS‐C and its severity. If this correlation is substantiated in prospective studies,^41^ this might have significant implications for the recognition or management of a multitude of inflammatory diseases.

## AUTHOR CONTRIBUTIONS

EAL and JEGC conceived and designed the study. JEGC, EAL, and JN undertook data collection and processing. JEGC undertook analysis and interpretation. EAL wrote the first draft with contributions from JEGC and JN. EAL undertook the literature review. EAL and DFK provided supervision. AK, AS, SA, SP, SS, JJG, DFK, and EAL contributed critical and expert subspecialty review, referred and followed up patients included within the manuscript, and reviewed the final manuscript. All authors gave approval of the final version.

## FUNDING INFORMATION

No specific funding was received from any bodies in the public, commercial, or not‐for‐profit sectors to carry out the work described in this article. This manuscript has not submitted or published in any other journal.

## CONFLICT OF INTEREST STATEMENT

No authors declare any conflicts of interest in relation to this manuscript.

## Supporting information


**Data S1:** Supporting information.

## Data Availability

The data that support the findings of this study are available on request from the corresponding author. The data are not publicly available due to privacy or ethical restrictions.
