# Supplementary material for: Vitamin D may influence disease course in PIMS‐TS/MIS‐C: An observational cohort study
Source: Pediatr Int. 2025 Oct 16;67(1):e70241. doi: 10.1111/ped.70241 (PMC12529462; doi:10.1111/ped.70241)
Supplement: Supplementary file 1 — Data S1: Supporting information. [file PED-67-e70241-s001.docx]

**Table of Contents for Supplementary Materials**

[Supplemental Figure 1: Correlation matrix of continuous variables 2](#_Toc205967697)

[Supplementary Figure 2: Relationships between laboratory values and demographics 3](#_Toc205967698)

[Supplementary Figure 3: Relationship of fever to biochemical and clinical findings 4](#_Toc205967699)

[Supplementary Figure 4: Directed acyclic graph demonstrating novel hypothecated roles for vitamin D status in PIMS-TS/MIS-C 5](#_Toc205967700)

[Supplementary methods: 6](#_Toc205967701)

[Supplementary table 1: Previous studies and reviews on clinical and laboratory outcomes and treatment of children with PIMS-TS/MIS-C 8](#_Toc205967702)

# Supplemental Figure 1: Correlation matrix of continuous variables

Heatmap shows spearman nonparametric correlation between all continuous variables. Values in the lower left half represent spearman r values (negative values (red) demonstrating inverse correlation, positive values in blue). P values are shown in the upper/right half. P values are shown, with those <0.15 in colour (from yellow – orange – red, with decreasing p value).

# Supplementary Figure 2: Relationships between laboratory values and demographics

Comparisons are shown between age and CRP (**A**), NT-pro-BNP (**B**), lowest lymphocyte count (**C**), D-dimer (**E**), ferritin (**F**) and WHO weight centile for age (**G**). The likely timing of COVID-exposure or first positive PCR result is shown against weight-for-age centile (**H**) and CRP (**J**). Lastly, we show the relationship between highest recorded ferritin and CRP throughout admission for all patients (**I**). For all plots, spearman correlation (r and p values) are shown. Line of fit results from simple linear regression with 95% confidence intervals shown.

# Supplementary Figure 3: Relationship of fever to biochemical and clinical findings

For all plots, spearman correlation (r and p values) are shown. Line of fit results from simple linear regression with 95% confidence intervals shown. P values shown in (G) represent separate Mann-Whitney tests.

Abbreviations: LOS – length of stay; CRP – C-reactive protein


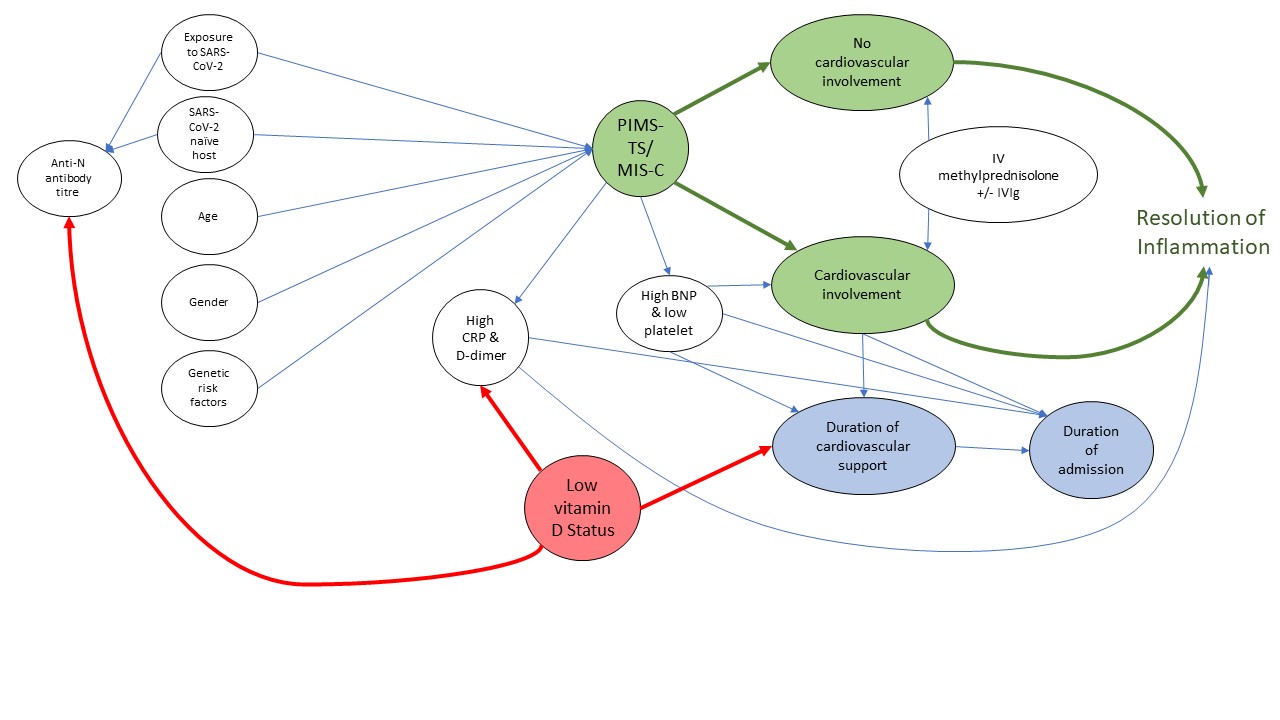


# Supplementary Figure 4: Directed acyclic graph demonstrating novel hypothecated roles for vitamin D status in PIMS-TS/MIS-C

Existing and known contributors to PIMS-TS/MIS-C are shown in black open circles with blue connecting lines, including selected markers of disease and disease intervention(s). The disease course including with and without cardiovascular involvement is followed with green circles and green connecting lines. Disease sequelae are shown by blue closed circles.

The hypothesized contribution of vitamin D status identified by this observational study are shown in red, including reduced anti-N titres, a relationship with higher CRP / D-dimer and a prolonged need for vasoactive medication. We confirm findings from others that IV methylprednisolone is effective in resolving markers of inflammation.

Abbreviations: CRP – C-reactive protein; MIS-C - Multisystem Inflammatory Syndrome in Children; NT- BNP – brain naturetic peptide; PIMS-TS - Paediatric Inflammatory Multisystem Syndrome Temporally Associated with SARS-CoV-2;

# Supplementary methods:

To compare continuous variables, we performed multiple Mann-Whitney U tests; where necessary this was controlled for multiple testing by applying a false discovery rate of 1%, with two-stage step-up method of Benjamini, Krieger and Yekutieli. Dichotomous outcomes were compared by Fisher’s exact test. To control for age, sex and weight centile, multiple logistic regression was used to assess for factors which may differ by the need for vasoactive medication. Due to the small numbers of patients, separate multiple logistic regression was performed for presenting features and laboratory results. For comparison between two sets of continuous variables, Spearman nonparametric correlation were performed, with r and p values reported. Simple linear regression was performed to illustrate fit of the data, with 95% confidence intervals shown. With CRP, all available data is reported with time 0 as the time from treatment starting (or admission where no immunomodulatory treatment was given). To illustrate the decline observed amongst sub-groups, thick lines are overlayed on the individual patient datasets. These are fitted to the data by lowess curve, using a coarse 5-point smoothing window.

Ethical review

The project was initially registered as a service evaluation of the paediatric infectious disease and immunology service within Oxford University Hospitals NHS Foundation Trust. Following collection of data, and internal presentation, clinicians (authors of this manuscript) were recruited to report anonymised patient data and report the outcomes of multiple domains. The UK Health Research Authority (UK HRA) orchestrate the UK National Health Service ethical approval applications (NHS REC) and arbitrate on where REC approval is required. In keeping with UK HRA advice, ethical approval is not required for anonymous reporting of patient data. Linked here, and cited below in full: <https://www.hra-decisiontools.org.uk/ethics/docs/Algorithm%20-%20Does%20my%20project%20require%20REC%20review%20v2.0%2020200304.pdf>

We quote from the UK HRA decision tool document:

*“****Research involving previously collected, non-identifiable information***

*Research limited to secondary use of information previously collected in the course of normal care (without an intention to use it for research at the time of collection) is generally excluded from REC review, provided that the patients or service users are not identifiable to the research team in carrying out the research.*

*This exception also applies to research undertaken by staff within a care team using information previously collected in the course of care for their own patients or clients, provided that data is anonymised in conducting the research. Research involving information which has been anonymised by an intermediary (such as NHS Digital) before its onward release to the researchers is excluded from REC review provided that there is a legal basis for the anonymisation.”*

# Supplementary table 1: Previous studies and reviews on clinical and laboratory outcomes and treatment of children with PIMS-TS/MIS-C

| **Article** | **Country** | **Study type** | **Total cases** | **Median age (yrs)** | **Presenting symptoms reported** | **Clinical outcomes reported** | **Laboratory outcomes reported** | **Treatments reported upon** |
| --- | --- | --- | --- | --- | --- | --- | --- | --- |
| Williams et al, 2020^1^ | USA, France, UK, Switzerland, Italy | Systematic review + meta-analysis | 833 | 9 | Fever (96%), gastrointestinal symptoms (85%), rash (58%), conjunctival injection (52%), respiratory symptoms (43%), oral mucosal changes (42%), peripheral extremity changes (39%), neurological symptoms (32%), cervical lymphadenopathy (24%), musculoskeletal symptoms (17%) | Cardiovascular complications included shock (65%), myocardial dysfunction (61%), myocarditis (65%), pericardial effusion (35%), and coronary artery abnormalities (39%), coronary artery aneurysm/dilatation (16%), admission to paediatric intensive care unit (PICU) (76%), mortality (2%) | SARS-CoV-2 antibody, PCR, C-reactive protein (CRP), D-dimer, procalcitonin, brain natriuretic peptide (BNP), fibrinogen, ferritin, troponin, interleukin 6 (IL-6), lymphocyte and platelet counts, albumin | Vasoactive medications (61%) and mechanical ventilation (25%). Treatment strategies included: intravenous immunoglobulin (IVIg; 82%), steroids (54%), steroids + IVIg (50%), IL-6 inhibitors (12.6%), IL-1Ra inhibitor (8.6%), antiplatelet drugs (64%), and anticoagulation (51%) |
| Sharma & Bhaskar, 2021^2^ | Pakistan, India, USA, Turkey, Saudi Arabia, Italy, Sweden, UK, Spain, Australia, Qatar | Systematic review + meta-analysis | 780 | 9 | Fever (100%), skin manifestations (64%), conjunctivitis (63%), respiratory distress (70%), gastrointestinal symptoms (90%), neurological manifestations (41%), | Coronary artery abnormalities (13%), shock (60%), admission to PICU (71%) | Haemoglobin, ferritin, CRP, d-dimer, neutrophil, lymphocyte and platelet counts, albumin, troponin | Vasoactive medications (54%), mechanical ventilation 49%. Treatment strategies included: IVIg (80%), steroids (70%), IL-blockers (12%), |
| Santos et al, 2022^3^ | USA, UK, India, Turkey, Iran, India, Switzerland, France, Belgium, Poland, Brazil, Italy, Ireland, Iran, Germany, Spain, Israel, Pakistan, Chile, S. Africa, Algeria, Czechia | Systematic review + meta-analysis | 2275 | 8.9 | Fever (100%), gastrointestinal symptoms (82%), cough (41%), headache (28%), conjunctivitis (54%), respiratory symptoms (39%), rash (59%), neurological symptoms (28%) | Shock (60%), cardiac symptoms (66%), admission to PICU (76%), mortality (1%) | SARS-CoV-2 antibody, PCR | Vasoactive medications (54%), mechanical / non-invasive ventilation (50%). Treatment strategies included: IVIg (84%), steroids (64%), biological immunomodulation (27%), antiplatelet therapy (78%) |
| Hoste, Van Paemel & Haerynck, 2021^4^ | UK, USA, France, India | Systematic review | 953 | 8.4 | Fever (99%), gastrointestinal symptoms (86%), respiratory symptoms (50%), exanthem (55%), non-purulent conjunctivitis (50%) | Tachycardia (77%), shock (60%), myocarditis (41%), decreased left ventricular function (40%), coronary aneurysm (10%), pericardial effusion (22%), PICU admission (73%), mortality (1.9%) | CRP, ferritin, IL-6, total white blood cell & lymphocyte count, sodium, d-dimers, platelets, troponin, BNP, SARS-CoV-2 antibody, PCR | Vasoactive medications (55%), mechanical/non-invasive ventilation (24%). Treatment strategies included: IVIg (75.9%), second IVIg dose (11%), corticosteroids (57%), IL-1R antagonist (8%), IL-6 inhibitor (7%), antiplatelet therapy (52%) |
| Awasthi et al, 2022^5^ | N. India | Case series | 40 | 7 | Fever (98%), mucocutaneous features (80%), abdominal symptoms (73%), respiratory symptoms (50%), non-purulent conjunctivitis (10%), digital gangrene (5%) | Myocardial dysfunction (73%), coronary artery dilatation or aneurysm (23%), shock (80%), PICU admission (85%), mortality (5%) | CRP, lymphocyte and platelet counts, procalcitonin, ferritin, d-dimer, fibrinogen, NT-proBNP, SARS-CoV-2 antibody, PCR | Vasoactive medications (73%), invasive ventilation (23%). Treatment strategies included: IVIg (100%), steroids (85%), antiplatelet therapy (80%) |
| Mannarino et al, 2022^6^ | Italy | Case series | 32 | 10 | Fever (100%), gastrointestinal symptoms (94%), rash (44%), conjunctivitis (56%), respiratory symptoms (41%), extremity changes (22%), oromucosal changes (25%), neurological symptoms (16%) | Shock (25%), PICU admission (69%), reduced left ventricular function (73%), mitral regurgitation (72%), coronary ectasia (3%), ECG anomalies (44%) | SARS-CoV-2 antibody, PCR, total white blood cell count (WBC), neutrophil, lymphocyte and platelet counts, haemoglobin, ferritin, CRP, troponin, NT-proBNP, d-dimer, IL-6 | Vasoactive medications (34%), non-invasive ventilation (38%). Treatment strategies included: IVIg (100%), methylprednisolone (94%), oral corticosteroid following IV treatment (94%), antiplatelet therapy (91%) |
| Ciftdogan et al, 2022^7^ | Turkey | Case series | 614 | 7.4 | Fever (100%), fatigue (82%), gastrointestinal symptoms (77%), conjunctival injection (50%), rash (54%), mucous membrane changes (43%), neurological symptoms (28%) | Hepatic failure (4%), acute kidney injury (5%), macrophage activation syndrome (4%), abnormal ECG 17%, PICU admission (31%), congestive heart failure (16%), myocarditis (5%), valvulitis (31%), pericarditis (8%), coronary artery dilatation (10%), aneurysm (1%), mortality 1.8%) | SARS-CoV-2 antibody, PCR, WBC, neutrophil, lymphocyte, platelet and eosinophil counts, haemoglobin, CRP, ESR, procalcitonin, ferritin, LDH, AST, ALT, albumin, sodium, urea, creatinine, triglyceride, troponin, BNP, NT-proBNP, fibrinogen, d-dimer, APTT, PT | Vasoactive medications (19%), mechanical/non-invasive ventilation (14%). Treatment strategies included: IVIg (93%), corticosteroids (84%), immunomodulation (6%), plasma exchange (2%), antiplatelet therapy (67%) |
| Kıymet et al, 2021^8^ | Turkey | Case series | 58 | 6 | Fever (100%), respiratory symptoms (47%), gastrointestinal symptoms (31%), rash (33%), desquamation (10%), conjunctivitis (36%), unilateral lymphadenopathy (10%), peripheral oedema (16%), headache (5%) | Tachycardia (28%), hypotension (21%), systolic dysfunction (14%), coronary artery involvement (5%), mitral regurgitation (40%)PICU admission (26%), | SARS-CoV-2 antibody, PCR, WBC, neutrophil, lymphocyte and platelet counts, ALT, AST, albumin uric acid, fibrinogen, d-dimer, CRP, procalcitonin, ESR, ferritin, creatinine | No comment on treatment |
| Solanki et al, 2022^9^ | E. India | Case series | 10 | 5.5 | Fever (100%), gastrointestinal symptoms (90%), rash (90%), conjunctivitis (70%), mucocutaneous changes (80%), encephalitis (10%) | Myocardial dysfunction (90%), hypotension (50%), mitral/tricuspid regurgitation (50%), PICU admission 100%, mortality (0%) | SARS-CoV-2 antibody, PCR, haemoglobin, neutrophil/lymphocyte counts, platelets, CRP, ESR, procalcitonin, ferritin, LDH, sodium, albumin, ALT, AST, calcium, urea, creatinine, d-dimer, fibrinogen, INR, amylase, triglycerides, troponin, CK-MB, BNP, myoglobin | Vasoactive medications (50%), non-invasive ventilation (40%). Treatment strategies: IVIg (80%), high-dose methylprednisolone (10-30mg/kg) (60%), low-dose methylprednisolone (3mg/kg) (40%), antiplatelet therapy (90%) |
| Mehra et al, 2021^10^ | Delhi, India | Cohort study | 120 | 7 | Fever (99%), gastrointestinal symptoms (71%), rash (66%), mucocutaneous involvement (74%), conjunctivitis (57%), respiratory distress (43%), encephalopathy (27%), seizures (5.8%) | LV dysfunction (31%), coronary abnormality (13%), pericardial effusion (13%), acute kidney injury (23%), PICU admission (90%), mortality (3.3%) | SARS-CoV-2 antibody, PCR, WBC, neutrophil/lymphocyte and platelet count, CRP, ferritin, d-dimer, ALT, INR, troponin-I, CPKMB, NT-ProBNP | Vasoactive medications (53%), invasive/non-invasive ventilation (43%), renal replacement therapy (8%). Treatment strategies: steroids (79%), IVIg (68%), antiplatelet therapy (49%) |
| Shabab et al, 2021^11^ | Michigan, USA | Case series | 26 | 8.7 | Fever (100%), gastrointestinal symptoms (85%), rash (58%), other features of Kawasaki disease (65%), | Abnormal ECG (tachy/bradycardia/non-specific ST-segment/T-wave changes, prolonged PR or QTc) (76%), coronary artery dilatation (Z-score >2) (46%), other cardiac change (reduced LV/RV function, MR/TR, aortic root dilatation, pericardial effusion (58%), PICU admission (57%), mortality (4%) | SARS-CoV-2 antibody, PCR, IL-2/IL-2R, IL-5, IL-6, IL-10, IL-13, IL-17, TNF-α, TNF-γ, WBC, haemoglobin, platelets, lymphocyte count, sodium, creatinine, albumin, ALT, AST, LDH, triglycerides, ESR, CRP, procalcitonin, ferritin, d-dimer, BNP, troponin, CK, fibrinogen, PTT, INR | Vasoactive medications (46%), invasive/non-invasive ventilation (39%). Treatment strategies: IVIg (96%), steroids (85%), IL-1R antagonist (50%), TNF-α inhibitor (4%), antiplatelet therapy (96%) |
| Salman et al, 2022^12^ | Turkey | Case series | 17 | 10 | Fever (100%), mucocutaneous involvement (12%), conjunctivitis (35%), rash (35%), abdominal pain (71%), acute abdominal surgery (18%), lymphadenopathy (24%), limb oedema (47%), headache (24%), dyspnoea (35%), neurological involvement (29%), sore throat (41%), myalgia (35%) | Arrhythmia (6%), impaired cardiac function (18%), valvular regurgitation (6%), PICU admission (59%), mortality (6%) | SARS-CoV-2 antibody, PCR, WBC, lymphocyte count, haemoglobin, platelets, sodium, ALT, CRP, procalcitonin, albumin, d-dimer, ferritin, ESR, fibrinogen, troponin | Vasoactive medications (24%), invasive/non-invasive ventilation (35%). Treatment strategies: IVIg (88%), second IVIg (59%), IVIg + methylprednisolone (77%), IL-6R antagonist (12%), IL-1R antagonist (6%), plasmapheresis (6%) |
| Harasheh et al, 2022^13^ | USA | Case series | 106 | 8.4 | Fever (98%), mucocutaneous changes (58%), peripheral extremity changes (13%), conjunctival injection (53%), rash (46%), cough (17%), dyspnoea (12%), abdominal pain (75%), chest pain (14%), myalgia (23%) | Coronary artery dilatation (7%), small aneurysm (2%), systolic dysfunction (30%)  PICU admission (75%), mortality (0%) | SARS-CoV-2 PCR, antibody, troponin-I, NT-BNP | Vasoactive medications (64%), invasive/non-invasive ventilation (60%). Treatment strategies: IVIg (99%), IL-1R antagonist (74%), steroids (44%), IL-6R antagonist (3%), antiplatelet therapy (96%) |
| Diaz et al, 2021^14^ | Chile | Case series | 66 | 8 | Shock (79%) | Mortality (4.6%) | SARS-CoV-2 PCR, antibody, CRP, procalcitonin, WBC, platelets, IL-6 | Vasoactive medications (79%), mechanical ventilation (49%). Review was comparing sepsis versus MIS-C and did not report on treatments. |
| Miller et al, 2022^15^ | USA | Case series | 4470 | 9 | Fever (100%), abdominal pain (69%), vomiting (67%), conjunctival injection (55%), rash (55%), diarrhoea (54%), hypotension (52%), headache (34%), myalgia (30%), cough (29%), shortness of breath (28%), mucocutaneous involvement (74%), neck pain (22%), chest pain (14%), periorbital oedema (12%), cervical lymphadenopathy (11%), altered mental status (11%), syncope (5%) | Cardiac dysfunction (31%), pericardial effusion/pericarditis (22%), coronary artery aneurysm/dilatation (17%), myocarditis (15%), arrhythmias (7%), congestive heart failure (5%), pneumonia (23%), pleural effusion (21%), acute kidney injury (19%), PICU admission (63%), mortality (0.8%) | SARS-CoV-2 PCR, antibody, CRP, lymphocyte, neutrophil and platelet counts, Troponin, BNP/NT-pro BNP, d-dimer, ferritin, IL-6, fibrinogen | Invasive/non-invasive ventilation (34%), vasoactive medications (45%). Treatment strategies: IVIg (84%), steroids (77%), immunomodulation (21%) |
| Miller et al, 2022^16^ | USA | Case series | 5670 | 9 | Mucocutaneous involvement (71%), other symptoms not reported on in detail. | Cardiac dysfunction (29%), pericardial effusion/pericarditis (19%), coronary artery aneurysm/dilatation (15%), myocarditis (12%), pneumonia (23%), pleural effusion (19%), mesenteric adenitis (31%), acute kidney injury (19%), PICU admission (58%), mortality (0.7%) | Troponin, BNP/NT-proBNP, lymphocyte, platelet counts. Fully vaccinated (3%), partially vaccinated (5%) | Vasoactive medications (41%), invasive/non-invasive ventilation (22%). Treatment strategies: IVIg (84%), steroids (81%) |
| Elilarasi et al, 2021^17^ | Chennai, India | Case series | 65 | 5 | Fever (97%), vomiting (72%), conjunctivitis (69%), oedema (55%), rash (54%), diarrhoea (45%), shortness of breath (32%), abdominal pain (50%), mucocutaneous changes (31%), altered sensorium (29%), myalgia (22%), headache (13%), sore throat (9%), desquamation (9%) | Hypotension (26%), shock (42%), coronary artery abnormalities (68%; most commonly small aneurysm), left ventricular dysfunction (25%), tachycardia (60%), pericardial effusion (49%), mitral regurgitation (26%), PICU admission (51%), mortality (6%) | SARS-CoV-2 PCR, antibody, haemoglobin, neutrophil and lymphocyte counts, platelets, urea, ALT, AST, bilirubin, sodium, albumin, LDH | Vasoactive medications (39%), respiratory support (54%). Treatment strategies: IVIg (18%), IVIg + methylprednisolone (55%), methylprednisolone/dexamethasone (14%), IL-6R antagonist (6%) |
| Stewart et al, 2022^18^ | UK | Case series | 110 | 10.2 | Fever (100%), respiratory distress (29%), cough (8%), abdominal pain (72%), diarrhoea (59%), vomiting (60%) | Acute kidney injury (30%), myocardial dysfunction (43%) PICU admission (89%) | SARS-CoV-2 PCR, antibody, triglycerides, ferritin, CRP, IL-6, d-dimer, NT-proBNP, troponin, sodium, phosphate, lymphocyte count, albumin, urea, creatinine, haematuria/proteinuria | Vasoactive medications (76%), invasive ventilation (20%). Treatment strategies: methylprednisolone (82%), IVIg (70%), IL-6R antagonist (5%), IL-1R antagonist (7%) |
| Kostik et al, 2021^19^ | Russia | Retrospective cohort study | 72 | 8.9 | Gastrointestinal symptoms (82%), neurological symptoms (45%), sore throat (75%), rash (82%), conjunctivitis (93%), mucosal changes (75%), respiratory symptoms (56%), cervical lymphadenopathy (76%), peripheral oedema (79%), desquamation (57%), facial swelling (62%), arthralgia (25%), CNS involvement (43%) | Myocarditis (47%), pericarditis (43%), coronary artery dilatation/aneurysm (18%), hypotension/shock (47%) | SARS-CoV-2 PCR, antibody, platelets, CRP, ALT, AST, LDH, creatinine, triglycerides, troponin, d-dimer | Treatments not reported upon |
| Khan et al, 2021^20^ | USA | Retrospective cohort study | 24 | 8.1 | Fever (90%), skin rash (62%), URTI symptoms (38%), GI symptoms (44%), LRTI symptoms (30%), arthralgia/myalgia (50%), swelling of extremities (24%), conjunctivitis (72%), encephalopathy (30%), mucosal involvement (68%) | ECG abnormalities (13%), coronary dilatation (8%), depressed systolic function (33%), shock (79%), PICU admission (66%), mortality (4.2%) | SARS-CoV-2 PCR, antibody, WBC, ESR, CRP, ferritin, d-dimer, LDH, procalcitonin, BNP, albumin, haemoglobin | Vasoactive medications (54%), invasive/non-invasive ventilation (13%). Treatment strategies: steroids (71%), IVIg (92%), IL-1R antagonist (17%), aspirin (42%) |
| Kahn et al, 2021^21^ | Sweden | Longitudinal study | 133 | 9.3 | Fever (99%), abdominal pain (70%), rash (51%), non-purulent conjunctivitis (54%), diarrhoea (44%), headache (42%), lethargy (30%), lymphadenopathy (20%), oedema (19%), sore throat (16%) irritability (12%), mucosal involvement (9%), dyspnoea (8%), encephalopathy (4%) | Reduced ejection fraction (19%), coronary artery involvement (16%), pericardial effusion (9%), arrhythmia (5%), PICU admission (16%) | SARS-CoV-2 PCR, antibody, ESR, albumin, troponin, NT-ProBNP | Treatments not commented upon |
| Acevedo et al, 2021^22^ | Colombia | Case series | 78 | 7 | Fever (100%), diarrhoea (71%), abdominal pain (71%), vomiting (74%), mucosal involvement (22%), peripheral oedema (44%), conjunctival injection (30%), headache (22%) | Shock/systolic myocardial dysfunction (78%), coronary artery aneurysms (Z-score >2.5, 36%), pericardial effusion (36%), mitral regurgitation (21%), arrhythmia (6%), acute kidney injury (29%), PICU admission (100%), mortality (9%) | SARS-CoV-2 PCR, antibody, troponin, pro-BNP, CRP, ESR, d-dimer, ferritin, haemoglobin, lymphocyte count, albumin, bilirubin | Vasoactive medications (76%), invasive ventilation (14%). Treatment strategies: IVIg (91%), steroids (71%), antiplatelet medications (44%) |
| Fabi et al, 2021^23^ | Italy | Case series | 24 | 6.8 | Conjunctival injection (54%), peripheral oedema (33%), rash (50%), mucosal changes (42%), cervical lymphadenopathy (13%), respiratory symptoms (25%), abdominal symptoms (88%) | Hypotension (33%), left ventricular dysfunction (46%), valvular dysfunction (42%), coronary artery abnormalities (46%, aneurysm 16%), arrhythmia (33%), PICU admission (46%) | SARS-CoV-2 PCR, antibody, WBC, lymphocyte and neutrophil counts, sodium, potassium, platelets, ESR, CRP, procalcitonin, IL-6, IL-10, IL-8, TNF-alpha, IL-1 beta, IL-12p&), d-dimer, ferritin, BNP, troponin-I, lymphocyte subsets | Vasoactive medications (54%). Treatment strategy: IVIg + steroids (96%), TNF-alpha inhibitor (4%), aspirin (100%) |
| Channon-Wells et al, 2023^24^ | Worldwide (39 countries) | Cohort study | 2009 | 8 | Fever (93%), sore throat (23%), cough (20%), respiratory distress (13%), abdominal pain (60%), diarrhoea (44%), vomiting (53%), headache (30%), irritability (18%), lethargy (33%) | Coronary aneurysm (12%), left ventricular dysfunction (13%), mortality (0.01%) | SARS-CoV-2 PCR, antibody, lymphocyte count, troponin, CRP, ferritin, albumin | Ventilation/vasoactive medications (27%). Treatment strategies: IVIg (34%), IVIg + glucocorticoids (34%), glucocorticoids (24%), other combination including biological therapy (2.9%), no treatment (4%) |
| de Farias et al., 2024^25^ | Brazil  (PICU only) | Case series | 79 | 1.9 | Shock syndrome associated to Kawasaki disease | Mortality (41.2%), lymphadenopathy, dyspnoea, GI symptoms, pneumonia, exanthema, cardiogenic shock, VIS (vasoactive-inotropic score), Oxygen low interfaces, invasive mechanical ventilation, non-invasive ventilation, ventilator weaning success, tracheostomy tube use, ARDS / severe, ARDS, MOD >2 organs, renal replacement, KDIGO stage 1, Length of stay hospital / PICU, mechanical ventilation time, ventilatory free days on day 28, PRISMIV%, PELOD-2, tidal volume, respiratory rate, peep-peak paw, driving pressure, compliance respiratory system, ventilatory ratio, oxygenation index, | Troponin I, platelet count, ESR, SARS-CoV-2 PCR, antigen and serology, PaO2/FiO2, PO2, sodium bicarbonate, anion gap, lactate, platelets, lymphocytes, INR, D-dimer, fibrinogen, CRP, ferritin, urea, creatinine, AST, ALT, albumin | Vasoactive inotropic support, respiratory support, IV methylprednisolone, IVIg, low molecular weight heparin, |
| Petrovic et al., 2023^26^ | Croatia | Case series | 25 (4 excluded) | 8 | Five mild (24%), eight moderate (38%), and eight (38%) severe MIS-C patients. Systolic dysfunction and symptomatic myocarditis were diagnosed in 33% of our patients. Most of the severe MIS-C patients (6; 75%) had cardiac involvement. We had no patients with coronary artery dilatation or aneurysm. | 33% of patients had significant cardiac involvement, coronary artery aneurysm (0%), (38%) had severe MIS-C and were admitted and treated in the PICU | hypovitaminosis D in almost all of our MIS-C patients (95% - median value 35.3 nmol/L), procalcitonin, CRP, ESR, leucocyte count, lymphocyte count, platelet count, erythrocyte count, fibrinogen, D-dimer, IL-6, ferritin, albumin, total proteins, triglycerides, liver enzymes, electrolytes, troponin T, and NT-pro-BNP |  |
| Darren et al., 2021^27^ | UK | Case series | 18 | 8.9 | All children who met the Royal College of Paediatrics and Child Health, Centers for Disease Control, and WHO case definitions of PIMS-TS. | Mortality (0%), PICU 67%, vasopressors/inotropes (67%), invasive ventilation (22%), haemofiltration (6%), LVEF | CRP, ESR, Vitamin D [median 18·2 (range 7·8–38) nmol/l], Bone profile, SARS-CoV-2 PCR, IgG, | IVIg, methylprednisolone, tocilizumab, infliximab, prednisolone |
| Toporco Rivera et al., 2022^28^ | USA | Case series | 31 | 8 | <21 years of age with fever, laboratory evidence of inflammation, and evidence of clinically severe illness requiring hospitalization, with multisystem ≥2 organ involvement, no alternative plausible diagnoses and either positive for current or recent SARS-CoV-2 infection; or exposure to a suspected or confirmed Covid-19 case within 4 weeks. | Need for inotropic support, need for mechanical ventilation, VA ECMO, mortality (0%), VIS score, LVEF, | Severe vitamin D deficiency (32%), SARS-CoV-2 PCR, IgG, Calcium, troponin, creatinine, GFR, | Mechanical ventilation, inotropic support, VA ECMO |
| Keleş et al., 2023^29^ | Turkey | Cohort study | 51 | 8.8 | The case definition of MIS-C was used, as defined by the Centers for Disease Control and Prevention and the World Health Organization |  |  |  |
| Mamishi et al., 2022^30^ | Iran | Case series | 122 | 6.4 | Nausea and vomiting (53.3%), skin rash (49.6%), abdominal pain (46.7%) and conjunctivitis (41.8%) were also frequently seen Headache, chest pain, tachypnea and respiratory distress were significantly more common in patients with severe MIS-C. | 79.5% mild-moderate MIS-C (of whom 17.% had KD overlap). 20.5% severe MIS-c. 60.7% male, 12.3% underlying disease, coronary artery dilatation 34.4%, Hypotension, myocarditis, valvulitis. Supplemental oxygen, 20.5% PICU, 1.6% intubated, 1.6% mortality, hospital LOS, renal involvement 3.3% | SARS-CoV-2 PCR, IgG, FBC, biochemistry, AST, ALT, PT, PTT, albumin, ESR, CRP, ferritin, IL-6, fibrinogen, D-dimer, CK, CPK-MB, troponin, LDH, amylase, lipase, triglycerides, vitamin D levels.  Vitamin D levels with severe MISC (8.5 ng/mL) significantly lower than mild-moderate (20.5 ng/mL) MIS-C (P = 0.05) | Pulse glucocorticoids for patients with moderate or severe illness and high-dose glucocorticoids for patients with mild illness. Glucocorticoids in 77.3%, IVIg in 18.3% |

**References:**

1. Williams V, Dash N, Suthar R, et al. Clinicolaboratory Profile, Treatment, Intensive Care Needs, and Outcome of Pediatric Inflammatory Multisystem Syndrome Temporally Associated with SARS-CoV-2: A Systematic Review and Meta-analysis. *J Pediatr Intensive Care.* 2022;11(1):1-12.

2. Sharma D, Bhaskar SMM. Prevalence of paediatric hyperinflammatory conditions in paediatric and adolescent hospitalized COVID-19 patients: a systematic review and meta-analysis. *APMIS.* 2022;130(2):101-110.

3. Santos MO, Goncalves LC, Silva PAN, et al. Multisystem inflammatory syndrome (MIS-C): a systematic review and meta-analysis of clinical characteristics, treatment, and outcomes. *J Pediatr (Rio J).* 2022;98(4):338-349.

4. Hoste L, Van Paemel R, Haerynck F. Multisystem inflammatory syndrome in children related to COVID-19: a systematic review. *Eur J Pediatr.* 2021;180(7):2019-2034.

5. Awasthi P, Kumar V, Naganur S, et al. Multisystem Inflammatory Syndrome in Children: Follow-Up of a Cohort from North India. *Am J Trop Med Hyg.* 2022;106(4):1108-1112.

6. Mannarino S, Raso I, Garbin M, et al. Cardiac dysfunction in Multisystem Inflammatory Syndrome in Children: An Italian single-center study. *Ital J Pediatr.* 2022;48(1):25.

7. Yilmaz Ciftdogan D, Ekemen Keles Y, Cetin BS, et al. COVID-19 associated multisystemic inflammatory syndrome in 614 children with and without overlap with Kawasaki disease-Turk MIS-C study group. *Eur J Pediatr.* 2022;181(5):2031-2043.

8. Kiymet E, Boncuoglu E, Sahinkaya S, et al. A Comparative Study of Children with MIS-C between Admitted to the Pediatric Intensive Care Unit and Pediatric Ward: A One-Year Retrospective Study. *J Trop Pediatr.* 2021;67(6).

9. Solanki R, Gupta A, Roy S, Pal S, Khan MF. The "After Wave": Pediatric multisystem inflammatory syndrome temporally associated with COVID-19 in a series of children from Eastern India. *Med J Armed Forces India.* 2022;78(Suppl 1):S133-S138.

10. Mehra B, Pandey M, Gupta D, et al. COVID-19-associated Multisystem Inflammatory Syndrome in Children: A Multicentric Retrospective Cohort Study. *Indian J Crit Care Med.* 2021;25(10):1176-1182.

11. Shabab J, Dubisky A, Singh A, Crippen M, Abulaban K, Aldrich A. A descriptive study on multisystem inflammatory syndrome in children in a single center in West Michigan. *Pediatr Rheumatol Online J.* 2021;19(1):172.

12. Salman H, Aslan N, Akcam M, et al. COVID-19-associated multisystem inflammatory syndrome in children: Experiences of three centres in Turkey. *Mod Rheumatol.* 2022;32(2):460-466.

13. Harahsheh AS, Sharron MP, Bost JE, et al. Comparison of First and Second Wave Cohorts of Multisystem Inflammatory Disease Syndrome IN Children. *Pediatr Infect Dis J.* 2022;41(1):e21-e25.

14. Diaz F, Bustos BR, Yagnam F, et al. Comparison of Interleukin-6 Plasma Concentration in Multisystem Inflammatory Syndrome in Children Associated With SARS-CoV-2 and Pediatric Sepsis. *Front Pediatr.* 2021;9:756083.

15. Miller AD, Zambrano LD, Yousaf AR, et al. Multisystem Inflammatory Syndrome in Children-United States, February 2020-July 2021. *Clin Infect Dis.* 2022;75(1):e1165-e1175.

16. Miller AD, Yousaf AR, Bornstein E, et al. Multisystem Inflammatory Syndrome in Children During Severe Acute Respiratory Syndrome Coronavirus 2 (SARS-CoV-2) Delta and Omicron Variant Circulation-United States, July 2021-January 2022. *Clin Infect Dis.* 2022;75(Suppl 2):S303-S307.

17. Elilarasi S, Poovazhagi V, Kumaravel G, Srividya VG, Solomon JRS. Pediatric Inflammatory Multisystem Syndrome Temporally Associated with SARS-CoV-2. *Indian J Pediatr.* 2022;89(9):879-884.

18. Stewart DJ, Mudalige NL, Johnson M, Shroff R, du Pre P, Stojanovic J. Acute kidney injury in paediatric inflammatory multisystem syndrome temporally associated with SARS-CoV-2 (PIMS-TS) is not associated with progression to chronic kidney disease. *Arch Dis Child.* 2022;107(3):e21.

19. Kostik MM, Bregel LV, Avrusin IS, et al. Distinguishing Between Multisystem Inflammatory Syndrome, Associated With COVID-19 in Children and the Kawasaki Disease: Development of Preliminary Criteria Based on the Data of the Retrospective Multicenter Cohort Study. *Front Pediatr.* 2021;9:787353.

20. Khan M, Dang L, Singh H, Dalrymple A, Miller A, Tanios A. Spectrum of SARS-CoV-2-Related Clinical Syndromes in Children: A Year in the Life. *Clin Pediatr (Phila).* 2022;61(2):188-193.

21. Kahn R, Berg S, Berntson L, et al. Population-based study of multisystem inflammatory syndrome associated with COVID-19 found that 36% of children had persistent symptoms. *Acta Paediatr.* 2022;111(2):354-362.

22. Acevedo L, Pineres-Olave BE, Nino-Serna LF, et al. Mortality and clinical characteristics of multisystem inflammatory syndrome in children (MIS-C) associated with covid-19 in critically ill patients: an observational multicenter study (MISCO study). *BMC Pediatr.* 2021;21(1):516.

23. Fabi M, Filice E, Biagi C, et al. Multisystem Inflammatory Syndrome Following SARS-CoV-2 Infection in Children: One Year after the Onset of the Pandemic in a High-Incidence Area. *Viruses.* 2021;13(10).

24. Channon-Wells S, Vito O, McArdle AJ, et al. Immunoglobulin, glucocorticoid, or combination therapy for multisystem inflammatory syndrome in children: a propensity-weighted cohort study. *Lancet Rheumatol.* 2023;5(4):e184-e199.

25. de Farias ECF, Pavao Junior MJC, de Sales SCD, et al. Factors associated to mortality in children with critical COVID-19 and multisystem inflammatory syndrome in a resource-poor setting. *Sci Rep.* 2024;14(1):5539.

26. Petrovic D, Benzon B, Srsen S, et al. The Impact of Vitamin D Levels on Clinical Manifestations of Multisystem Inflammatory Syndrome in Children: A Cross-Sectional Study. *Life (Basel).* 2023;13(3).

27. Darren A, Osman M, Masilamani K, et al. Vitamin D status of children with paediatric inflammatory multisystem syndrome temporally associated with severe acute respiratory syndrome coronavirus 2 (PIMS-TS). *Br J Nutr.* 2022;127(6):896-903.

28. Torpoco Rivera D, Misra A, Sanil Y, Sabzghabaei N, Safa R, Garcia RU. Vitamin D and morbidity in children with Multisystem inflammatory syndrome related to Covid-19. *Prog Pediatr Cardiol.* 2022;66:101507.

29. Ekemen Keleş Y YD, Taşar S, Üstündağ G, Şahin A, Tuz AE, Arslan Maden A, Kara Aksay A, Çolak A, Karadağ Öncel E. . Can Serum 25-Hydroxy Vitamin D Levels Predict the Severity of Multisystem Inflammatory Syndrome in Children and COVID-19? . *Clin Res Pediatr Endocrinol.* 2023;29:190-198.

30. Mamishi S, Olfat M, Pourakbari B, et al. Multisystem inflammatory syndrome associated with SARS-CoV-2 infection in children: update and new insights from the second report of an Iranian referral hospital. *Epidemiol Infect.* 2022;150:e179.
